# Characterization of genetics in patients with mucosal melanoma treated with immune checkpoint blockade

**DOI:** 10.1002/cam4.3789

**Published:** 2021-03-15

**Authors:** Elizabeth I. Buchbinder, Jason L. Weirather, Michael Manos, Brian J. Quattrochi, Lynette M. Sholl, Ryan C. Brennick, Peter Bowling, Nancy Bailey, Lisa Magarace, Patrick A. Ott, Rizwan Haq, Benjamin Izar, Anita Giobbie‐Hurder, F. Stephen Hodi

**Affiliations:** ^1^ Department of Medical Oncology Dana‐Farber Cancer Institute Boston MA USA; ^2^ Department of Medicine Brigham and Women’s Hospital Boston MA USA; ^3^ Harvard Medical School Boston MA USA; ^4^ Department of Pathology Brigham and Women’s Hospital Boston MA USA; ^5^ Center for Immuno‐Oncology Dana‐Farber Cancer Institute Boston MA USA; ^6^ Broad Institute of MIT and Harvard Cambridge MA USA; ^7^ Division of Biostatistics Department of Data Sciences Dana‐Farber Cancer Institute Boston MA USA

**Keywords:** genetics, immune checkpoint blockade, immunotherapy, KIT mutation, mucosal melanoma

## Abstract

Mucosal melanoma is a rare form of melanoma which arises from melanocytes in the mucosal membranes and can be effectively treated with immune checkpoint blockade (ICB). However, response rates in mucosal melanoma are lower than those observed for cutaneous melanomas.

Targeted sequencing of up to 447 genes (OncoPanel) was performed on tumors from all mucosal melanoma patients seen at the Dana‐Farber Cancer Institute from 2011 until March 2019.

We identified a total of 46 patients who received ICB with both tumor‐genotype and ICB response data available. Within this cohort of patients, 16 (35%) had durable clinical benefit (DCB) to their first line of ICB. The average mutational burden/megabase was 6.23 and did not correlate with tumor response to ICB. Patients with *KIT* aberrations had a higher DCB rate compared with patients with wildtype *KIT* (71 vs. 28%), but this was not found to be statistically significant. For comparison, we analyzed tumor genotypes from an additional 50 mucosal melanoma tumors and 189 cutaneous melanoma tumors. The most frequent mutations in mucosal melanoma were in *SF3B1* (27%), *KIT* (18%), and *NF1* (17%), a pattern that is distinct from cutaneous melanomas. In addition, there were genetic differences observed based upon the site of origin of the mucosal melanoma.

Our findings explore clinical features of response in patients with mucosal melanoma treated with ICB and demonstrate a low mutational burden that does not correlate with response. In addition, the lack of significant association between the genetic aberrations tested and response to ICB indicates the need for further exploration in this patient population.

## Graphical abstract

Mucosal melanoma is a rare form of melanoma that arises from mucosal membranes within the nose, intestines or vaginal cavity. It can be treated with new immunotherapies that target immune checkpoints with some success but not all patients respond to these therapies. This study examines the genetic changes within mucosal melanoma to look for patterns that may help predict who will respond well to immunotherapy and who will not respond. Some interesting trends were observed but more work is needed to determine genetic mutations in mucosal melanoma that are associated with treatment.

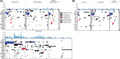

## 1 BACKGROUND

The treatment of cutaneous melanoma has advanced dramatically over the last several years with the introduction of immune checkpoint blockade.^1^, ^2^ A subset of melanomas (1.5%) arise from melanocytes that are localized in mucous membranes leading to mucosal melanoma.^3^ Mucosal melanomas have high rates of recurrence after initial surgery and are associated with poor prognosis at recurrence.^4^, ^5^ Immune checkpoint blockade (ICB) has demonstrated efficacy in the treatment of mucosal melanoma with a response rate of 19–23% to single agent PD‐1 inhibition compared with a 40% response rate in patients with cutaneous melanoma.^6^, ^7^, ^8^ Combined ICB with PD‐1 and CTLA‐4 inhibition has also demonstrated efficacy in the treatment of mucosal melanoma albeit at a lower rate compared with cutaneous melanoma (37% vs. 55–60%).^7^ Responses to immunotherapy vary by subtype of melanoma with high response rates observed in desmoplastic melanomas that are felt to be related to high mutational burden and pre‐existing immune response.^9^

Although the responses to immune checkpoint blockade in patients with mucosal melanoma are encouraging, the differences between mucosal melanoma and cutaneous melanoma make this an interesting population to explore further. Mutational load has previously been associated with response to immunotherapy.^10^, ^11^, ^12^ Mucosal melanomas do not arise in UV‐exposed areas and therefore a lower mutational burden compared with cutaneous melanoma would be expected. Previous characterizations of melanoma subtypes have demonstrated lower mutational burden in acral/mucosal melanoma with a mean of 2.64 mutations per megabase compared with a mean of 101 observed in cutaneous melanoma.^13^ When compared to other cancers with low mutational burden, the response rate in mucosal melanoma is higher than would be expected based upon this factor alone.

A better understanding of the genetic landscape of mucosal melanomas and how it relates to response to ICB may help guide care and assist in our understanding of immunotherapy. Previous genetic analyses have uncovered increases in *KIT* mutations in mucosal melanoma. These studies have also demonstrated the presence of *NF1*, *SF3B1*, and *NRAS* mutations in mucosal melanoma.^13^, ^14^, ^15^, ^16^, ^17^ Studies have also looked at *CDK4*, *MDM2*, *TERT*, and *AGAP2*.^18^, ^19^ In this study, we expand upon these previous explorations of the genetic profile of mucosal melanoma by correlating it with response to ICB.

## 2 METHODS

Tumor genotyping was performed by Oncopanel analysis on all mucosal melanoma patients seen by medical oncology within the Dana‐Farber Cancer Institute (DFCI) from 2011 until March 2019.

Patients were identified through the DFCI Oncology Data Retrieval System (OncDRS).^20^ OncDRS was used for the aggregation, management, and delivery of the clinical and operational research data used in this project.

Tumor genotyping was performed by OncoPanel.^21^, ^22^, ^23^, ^24^ OncoPanel is a cancer genomic assay to detect somatic mutations, copy number variations, and structural variants in tumor DNA extracted from fresh, frozen, or formalin‐fixed paraffin‐embedded samples. The OncoPanel assay surveys exonic DNA sequences of up to 447 cancer genes and 191 regions across 60 genes for rearrangement detection. DNA is isolated from tissue containing at least 20% tumor nuclei and analyzed by massively parallel sequencing using a solution‐phase Agilent SureSelect hybrid capture kit and an Illumina HiSeq 2500 sequencer. Genes targeted by OncoPanel are updated periodically to include additional targets, and spike‐in hybridization probes have been included to improve structural variant detection. The OncoPanel assay provides reports on single‐nucleotide variants, copy number variants, structural variants, tumor mutation burden (TMB), and select mutational signatures (e.g., UV exposure, smoking). Germline variants were removed bioinformatically using a panel of normal samples; variants present at >=0.1% frequency in publicly available databases were filtered (gnomad; Broad institute). All OncoPanel results were reviewed by molecular pathologists, and variants were tiered according to therapeutic, prognostic, and biologic relevance, as previously described (Sholl, et al JCI Insight 2016; Garofalo et al Genome Medicine 2016). Additional genetic data were obtained from public data available through cBioPortal for tumor‐site comparison purposes.^25^ The genetic data obtained for non‐DFCI patients was from IMPACT.^26^ To determine whether there may be some bias in variant calls or tumor genetics between DFCI and MSK treatment centers, we tested for a non‐random distribution of variants among the 183 genes common to all panels. No gene variants had a significant non‐random distribution with treatment centers (Fisher's exact test, *α* = 0.05).

After initial unsupervised analysis comparing mutational rate to tumor site for each gene, single‐nucleotide variants were manually curated by pathologist review to include only pathogenic or likely pathogenic variants in subsequent analyses, as determined by annotations in ClinVar or OncoKB.

Clinical data were obtained for the 46 patients treated at DFCI to evaluate site of disease, response to ICB, duration of response, progression free survival, and survival. As these patients were treated off study, the clinically meaningful endpoint of 6‐month durable clinical benefit rate (DCB) was chosen for the efficacy analysis. Clinical outcomes assessed were clinical response using RECIST 1.1 criteria, durable clinical benefit (DCB), progression‐free survival (PFS), overall survival (OS), duration of response (DOR), duration of stable disease (DSD), and duration of disease control (DDC). DCB is defined as lack of disease progression for at least 6 months after the initiation of treatment. PFS is defined as the time from first treatment to the earlier of disease progression or death. The follow‐up of patients who neither progressed nor died is censored at the date of last clinical visit. Overall survival is the time from first treatment to death from any cause. The follow‐up of patients alive is censored at the last assessment of vital status. DOR is defined for patients who achieved complete or partial response as best response to therapy and is defined as the interval between dates of first documentation of objective response and first documentation of progressive disease. In the absence of documented progressive disease, follow‐up was censored at date of last disease assessment. DSD and DDC are based on the date of the first scan. DSD is defined as the time interval until disease progression or death for patients with SD at the first scan. DDC is defined as the time until progression or death for patients with CR, PR, or SD at the time of the first scan. For both DDC and DSD, in the absence of disease progression or death, follow‐up was censored at the date of the last disease assessment.

The distributions of the time‐to‐event endpoints were based on the method of Kaplan–Meier. Median times are summarized with 95% confidence intervals estimated using log(‐log) methods.

Relationships between tumor genotype and either clinical outcome (DCB) or disease site (divided between Anal/rectal, Vulvovaginal, or Sinus/Nasopharynx origin) were assessed using the Fisher's exact test to test for non‐random overlap or mutual exclusion between variables. To correct for multiple tests, a null distribution was generated by shuffling either the clinical outcome (DCB), or disease site (*n* = 5000 permutations). An adjusted *p*‐value was calculated as the fraction of permutations where the smallest Fisher's exact test *p* value among 183 genes shared across the panels was less than or equal to the observed *p* value. This adjusted *p*‐value is indicates the overall expected false discovery rate accounting for multiple hypothesis testing, and thus an *α* = 0.1 will be regarded significant.

The association between tumor mutation burden and clinical outcome (DCB) was assessed by a two‐tailed Mann–Whitney U test. Comparisons of patient characteristics according to DCB were based on Wilcoxon rank‐sum (continuous) or Fisher's exact (categorical) tests.

## 3 RESULTS

### 3.1 Patient characteristics

A total of 65 mucosal melanoma patients who had tumor genotyping were identified; 63 underwent analysis with a 275, 300 or 447 gene panel, whereas two patient's tumors characterized by an older 41‐gene genotyping panel were excluded from further analysis. Of these 63 genotyped patient tumors, 46 received immunotherapy and had response data available.

For the genetic analysis all 63 genotyped patients were included. In addition, an additional 50 patients with mucosal melanoma and, for comparison, 189 patients with cutaneous melanoma were identified from public data available through cBioPortal from Memorial Sloan Kettering Cancer Center.^25^, ^26^ These were included in the genetic analysis but excluded from the analysis of clinical response.

Of the 46 mucosal melanoma patients for whom both genetic and clinical data were available, 78% were female and 22% were male. The primary site of the melanoma was anal/rectal in 35% of patients, vulvovaginal in 41%, and sinus/nasopharynx in 22%; one patient had a primary tumor that was either anal/rectal or vulvovaginal. Those with vulvar melanoma were only included if pathology or oncology classified the patient as a mucosal melanoma. The genetic testing was performed on the primary tumor in 32 of the patients and on a metastatic lesion in 14. As first line immunotherapy, 17% of patients received CTLA‐4 inhibition alone, 46% received PD‐1 (20) or PD‐L1 (1) inhibition alone, and 37% received combination therapy (Table 1). Immunotherapy was the first‐line systemic therapy in these patients.

**TABLE 1:** Summary of patient demographics and disease characteristics for the patients included in the response analysis

	All	Durable clinical benefit				*p*‐value
		No DCB		DCB		
	*N*	*N*	%	*N*	%	
Gender	36	23	63.9	13	36.1	0.99
Female						
Male	10	7	70.0	3	30.0	
Mean Age at diagnosis	**60.9**	**58.1**		**66.1**		**0.07**
Mean age at start of treatment	**62.5**	**60.0**		**67.0**		**0.12**
Primary Site	16	11	68.8	5	31.3	0.36
Anal/rectal						
Anal/rectal and vulvovaginal	1	—	—	1	100.0	
Sinus/nasopharynx	10	8	80.0	2	20.0	
Vulvovaginal	19	11	57.9	8	42.1	
Stage	21	13	61.9	8	38.1	0.61
M0						
M1B	11	6	54.5	5	45.5	
M1C	12	9	75.0	3	25.0	
M1D	2	2	100.0	—	—	
CPI Class	8	6	75.0	2	25.0	0.92
CTLA4						
Combination	17	11	64.7	6	35.3	
PD−1/PD‐L1	21	13	61.9	8	38.1	
LDH	6	3	50.0	3	50.0	0.41
Elevated (>231)						
Not elevated	40	27	67.5	13	32.5	

### 3.2 Clinical responses to treatment with ICB

**FIGURE 1:**
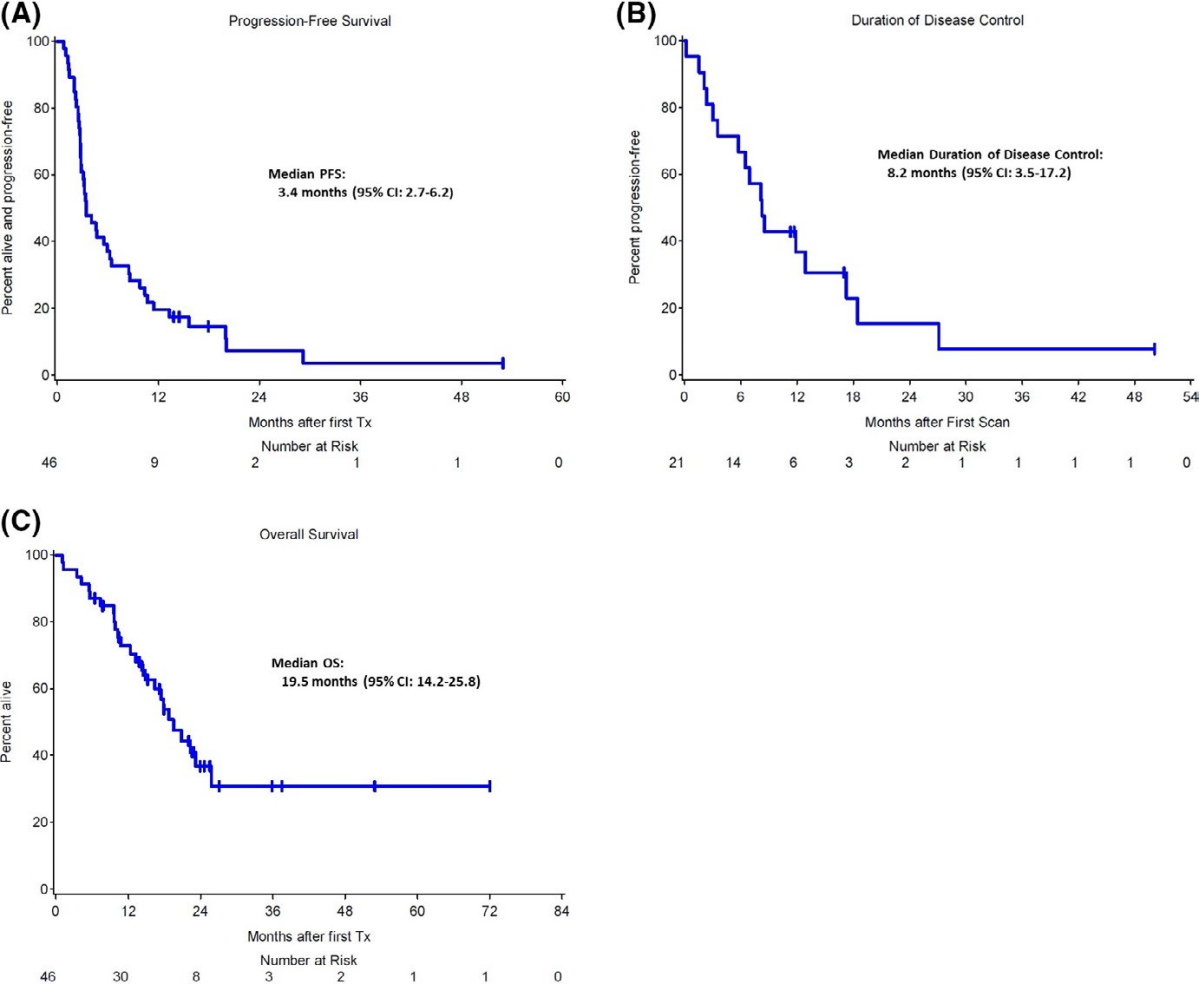
(A) Progression‐Free Survival (PFS), and Overall Survival (OS) in months after first immunotherapy for entire DFCI mucosal melanoma cohort. (B) Duration of disease control (DDC)

Median follow‐up was 23.9 months (inverted Kaplan‐Meier). Of the 46 mucosal melanoma patients who received ICB and had genetic analysis, two (4.4%) had complete response, seven (15.2%) had partial response, 12 (26.1%) had stable disease, and 25 (54.4%) had disease progression as best response to therapy. Sixteen (35%) had durable clinical benefit (DCB) to their first‐line IO therapy with a median duration of disease control of 8.2 months (95% CI: 3.5–17.2). Median progression‐free survival was 3.4 months (95% CI: 2.7–6.2) and median overall survival was 19.5 months (95% CI: 14.2–25.8). (Figure 1A). No differences in PFS or OS were seen between different classes of ICB or when combination therapy was given. The DCB rate in the CTLA‐4 arm was 25% (2 of 8) compared with 35% in combination (6 of 17) and 38% (8 of 21) in the PD‐1/PD‐L1 monotherapy (Data S1). Given the lack of difference in response rates to single versus combination therapy, the different ICB treatments were combined for the analysis. Two patients had objective responses to second‐line ICB; however, these responses were not durable and did not influence the analysis when included. Therefore, all data are based on the first exposure to ICB. We further explored the patterns of response to determine the duration of disease control (Figure 1B). The data suggest that patients with DCB were older at the time of diagnosis (median age: 66 vs. 56 years, *p* = 0.07). There were no differences in LDH between the two groups.

Ten of the patients on study received radiation therapy on the study. The majority of radiation was done concurrent with or sequential to immunotherapy. There was one patient with a PR who had vulvovaginal melanoma, there were three patients with SD (two with vulvovaginal disease and one sinus), six patients with progressive disease (3 sinus, 2 vulvovaginal, and one anal). Radiation did not appear to have an impact on ICB responsiveness in this cohort.

Further treatment beyond first‐line ICB was also recorded, 35% (16/46) of patients received no subsequent therapy. Of those patients who received subsequent therapy the majority received immunotherapy on clinical trial and/or combination immunotherapy. Three patients with *KIT* mutations received imatinib as a later line of therapy without documented responses to therapy (2 patients progressed and one patient had to stop due to toxicity from the therapy).

### 3.3 Genetic profiling of mucosal tumors and association with clinical response to ICB

**FIGURE 2:**
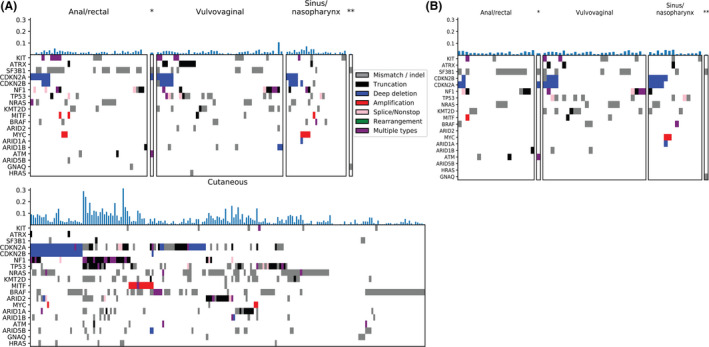
Mutational patterns in mucosal melanoma (top panels, *anal/rectal or vulvovaginal, **periorbital) vary by primary location. (A) entire cohort, (B) DFCI patients only. Mutational patterns observed in mucosal melanoma vary from those observed in cutaneous melanoma (bottom panel). The y‐axis of the color map lists genes that are mutated in 8% or more mucosal melanoma cases or are reported genes of interest in mucosal or cutaneous melanoma. Tumor mutation load (fraction of measured genes with alteration) is displayed for each patient (x‐axis) above the color map

**FIGURE 3:**
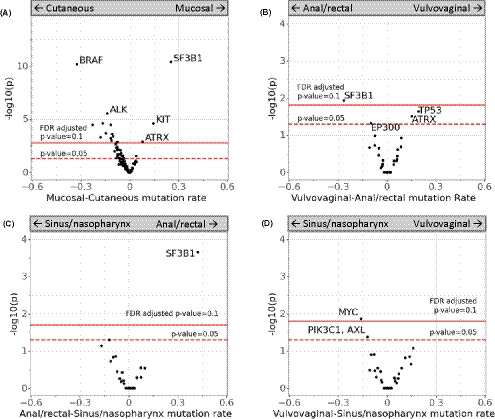
(A) Common mutations in mucosal melanoma vary from those observed in cutaneous melanoma, (B‐D) within mucosal melanoma samples, sites of disease also differed in variant composition between anal/rectal, vulvovaginal, and sinus/nasopharynx cases. The y‐axis indicates the negative logtransformed *p*‐value of the Fisher's exact test between variant/non‐variant counts for each gene between the disease sites (higher is more significant), with thresholds for the individual nominal *p*‐values indicated by a dotted line at alpha=0.05. The FDR‐adjusted *p*‐value at alpha=0.1 is shown as a solid line. The x‐axis indicates the relative difference in the frequency of the mutation between the sites described in the panel

The most frequent mutations seen in mucosal melanomas were in *SF3B1* (27%), *KIT (*18%), and *NF1* (17%). Three genes were enriched for mutations among mucosal melanomas in contrast to cutaneous melanomas: *SF3B1* (27% vs. 2%), *KIT* (18% vs. 3%), and *ATRX* (9% vs. 1%). These mutations have been observed previously in mucosal melanoma, particularly *KIT* and *SF3B1* mutations.^14^, ^15^ As expected, tumors from patients with cutaneous melanoma patients had higher TMB as compared with tumors from patients with mucosal melanoma (Figure 2, Figure 3A).

A broad array of *KIT* mutations and amplifications were observed, similar to what has been seen in the past in melanoma^27^ Mutations observed included activating mutations in the tyrosine kinase domain (D816 V) on exon 8and mutations in exon 11 and 13 (*W557R*, *L576P*, *K642E*).^28^

Mutations observed within mucosal melanomas varied by site of origin of the mucosal melanoma. A higher percentage of *SF3B1* mutations was observed in patients with melanoma of anal/rectal origin when compared with patients with vulvovaginal or nasopharynx melanoma. Patients with vulvovaginal melanoma had higher percentages of *TP53* mutations, which frequently correlated with *ATRX* mutations. In addition, a higher percentage of *MYC* mutations was observed in patients with sinus/nasopharynx melanoma when compared with patients with vulvovaginal melanomas (Figure 3B–D).

**FIGURE 4:**
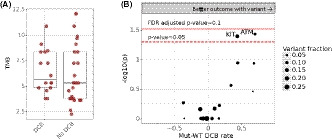
(A) Mutational load in immunotherapy in patients with and without durable clinical benefit (DCB). (B) Correlation between specific mutations and durable clinical benefit rate for the DFCI mucosal melanoma cohort, *ATM* (adjusted *p*‐value =0.15), *KIT* (adjusted *p*‐value =0.16). The y‐axis indicates the negative log‐transformed pvalue of the Fisher's exact test between variant/non‐variant counts for DCB and non‐DCB groups (higher is more significant), with thresholds for the individual nominal pvalues indicated by a dotted line at alpha=0.05. The FDR‐adjusted *p*‐value at alpha=0.1 is shown as a solid line. The x‐axis indicates the relative difference in the rate of clinical benefit between cases with and without a variant for each respective gene; only genes altered in at least 10% of cases are displayed

The average mutational burden/megabase for mucosal melanomas was 6.23 (95% CI: 3.63–10.89) and did not correlate with response (Figure 4A). Patients whose mucosal melanomas harbored a *KIT* mutation had one of the strongest associations with a favorable DCB, although it did not meet our threshold for statistical significance (71% vs. 28%, adjusted *p*‐value 0.16). *ATM* variants also had an association with a favorable DCB that similarly did not meet statistical significance. (Figure 4B) In regards to the two complete responses observed, one patient had none of the main mutations observed and the second had an *SF3B1* mutation. Both received combination immunotherapy.

## 4 DISCUSSION

In this study, we performed genetic profiling and assessed clinical responses to ICB in a cohort of patients with mucosal melanoma. Consistent with previous observations, we found a low mutational burden in this rare subset of melanoma. There was no correlation between TMB and response in this cohort of patients. We also did not observe significant correlations between the genetic variants tested and clinical response to ICB. There were no clinical differences between the groups to account for differences in response to immunotherapy. Interestingly, the response rate to PD‐1/PD‐L1 therapy alone was quite high in our cohort with similar response rates to combination immunotherapy.

The most common mutation in mucosal melanoma is in the *KIT* gene with a range of mutations and gene amplification observed. These mutations are activating mutations that lead to increased c‐kit signaling, thereby causing tumorigenesis.^29^, ^30^ C‐kit inhibitors have demonstrated modest benefit in clinical trials of patients with *KIT*‐mutated melanoma, but have been less effective in unselected cohorts of mucosal melanoma patients.^27^, ^31^, ^32^ Although there has been some work looking at combining c‐kit inhibition with immunotherapy in GI stromal tumors, the role that *KIT* mutation plays in sensitivity to immunotherapy remains unexplored.^33^ Further clinical trials are underway combining c‐kit inhibition with ICB and include patients with melanoma (NCT02571036). Our study supports this approach by suggesting that patients with *KIT* mutations might be more likely to benefit from immunotherapy, although this did not reach statistical significance. In this cohort, three patients received imatinib following immunotherapy and unfortunately no responses were observed to this.

*ATM* variants also trended toward a benefit to immunotherapy within our cohort. The role of ATM in DNA repair and cell cycle checkpoint may suggest a possible mechanism by which it leads to increased genetic instability and possibly immune response.^34^ However, this too needs further exploration.

While not associated with response to ICB, several mutations are associated with different primary sites of mucosal melanoma. A higher percentage of *SF3B1* mutations was observed in patients with melanoma of anal/rectal origin when compared with patients with vulvovaginal or nasopharynx melanoma. Splicing Factor 3b subunit 1 (*SF3B1*) encodes for one component of a complex involved in splicing mRNA, and mutation of *SF3B1 *has been observed to lead to alternate splicing.^35^ The differences in splicing when *SF3B1* is mutated may lead to varying gene expression and may vary based upon the tissue of origin since splices occur after transcription.^36^, ^37^ Another cancer with high rates of *SF3B1* mutations is uveal melanoma, which is relatively unresponsive to ICB.^38^, ^39^ In uveal melanoma, *SF3B1* mutations are associated with low‐grade disease and a favorable prognosis, whereas their role in mucosal melanoma is not clear to date.

In this cohort patients with vulvovaginal melanoma had higher percentages of *TP53* mutations, which frequently correlated with *ATRX* mutations. Mutations in the *ATRX* gene, which is involved in chromatin remodeling, have been observed in mucosal melanomas previously.^13^ Prior studies also found that mutations in *ATRX* frequently co‐occur with *TP53* mutations and are mutually exclusive to *SF3B1* mutations. As newer agents targeting splicing and chromatin remodeling are developed, the clinical patterns of mutations in mucosal melanoma may help guide trial selection.

We also examined the patterns of response to ICB for mucosal melanoma and found that response rate and disease control rate were consistent with previous reports.^6^, ^7^ As mentioned previously, the one difference in our cohort was higher than expected responses to PD‐1/PD‐L1 alone. This may have been influenced by the sample size or patient selection with better prognosis patients receiving single‐agent therapy. However, the majority of responses were not durable, which is distinct from what is generally expected with ICB in cutaneous melanoma.

Our study was limited due to the limited sample size due to the rarity of mucosal melanoma in the population. However, it expands upon previous studies of mucosal melanoma genetics by exploring the intersection between genetics and response to immunotherapy.^17^, ^19^

This analysis demonstrates the heterogenous role that genetics play in response to immunotherapy. At this time the role that NGS and TMB will play in treatment selection for mucosal melanoma is still being determined. However, genetic analysis and exploration of the immune microenvironment of this interesting subset of melanoma patients is just beginning. Additional testing will help to further understand the genetics, tumor microenvironment or other factors that explain the patterns of response to ICB observed. These studies provide an opportunity to guide cancer therapy for mucosal melanoma as well as other malignancies that are less responsive to immunotherapy.

## CONFLICT OF INTEREST

EB has served on advisory boards for Array Biopharma, Bristol‐Myers Squibb (BMS), Trieza Therapeutics and Novartis, and she also receives clinical trial support from Eli Lilly, Novartis, BMS, Genentech, and BVD. PAO reports the following: advisory roles for Alexion, Array, BMS, Celldex, CytomX, Genentech, Merck, Neon Therapeutics, Novartis, Pfizer, and TRM Oncology; institutional grants from Armo Biosciences, AstraZeneca/MedImmune, BMS, Celldex, CytomX, Genentech, Merck, Neon Therapeutics, Novartis, and Pfizer; and a speaking engagement from Medscape. SH reports the following: grants from BMS and Novartis; personal fees from BMS, Merck, Serono, Novartis, Takeda, Surface Pharmaceuticals, Genentech/Roche, Compass Therapeutics, Apricity, Bayer, Aduro, Partners Therapeutics, Sanofi, Pfizer, Pionyr Immunotherapeutics, 7 Hills Pharma, Verastem Oncology, Rheos Medicines, and Kairos Therapeutics; equity in Torque Therapeutics; and patents #20100111973 and #7250291 issued as well as #20170248603, #20160340407, #20160046716, #20140004112, #20170022275, #20170008962, and “Methods of Using Pembrolizumab and Trebananib” pending. L. Sholl‐ honorarium from Astra Zeneca; consulting income from EMD Serono; research grant funding from Roche/Genentech.

## ETHICS APPROVAL AND CONSENT TO PARTICIPATE

The study was approved by the institutional review board at Dana‐Farber/Harvard Cancer Center.

## Supporting information

Figs. S1‐S3

Data S1

## Data Availability

The data that support the findings of this study are available from the corresponding author upon reasonable request.
