# Supplementary material for: Characterization of genetics in patients with mucosal melanoma treated with immune checkpoint blockade
Source: Cancer Med. 2021 Mar 15;10(8):2627–35. doi: 10.1002/cam4.3789 (PMC8026918; doi:10.1002/cam4.3789)
Supplement: Supplementary file 1 — Figs. S1‐S3 [file CAM4-10-2627-s002.pptx]

## Slide 1
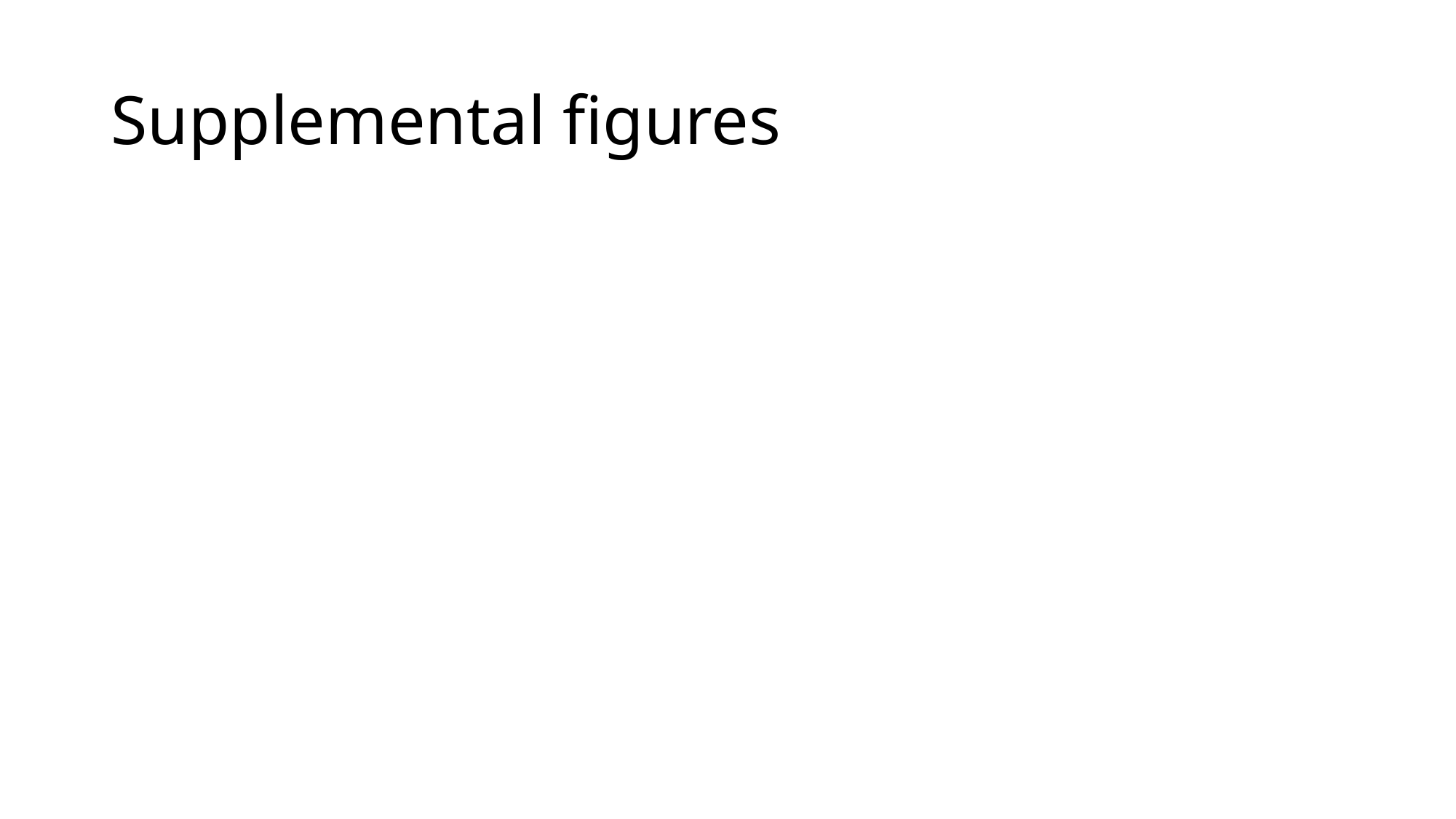

# Supplemental figures

## Slide 2
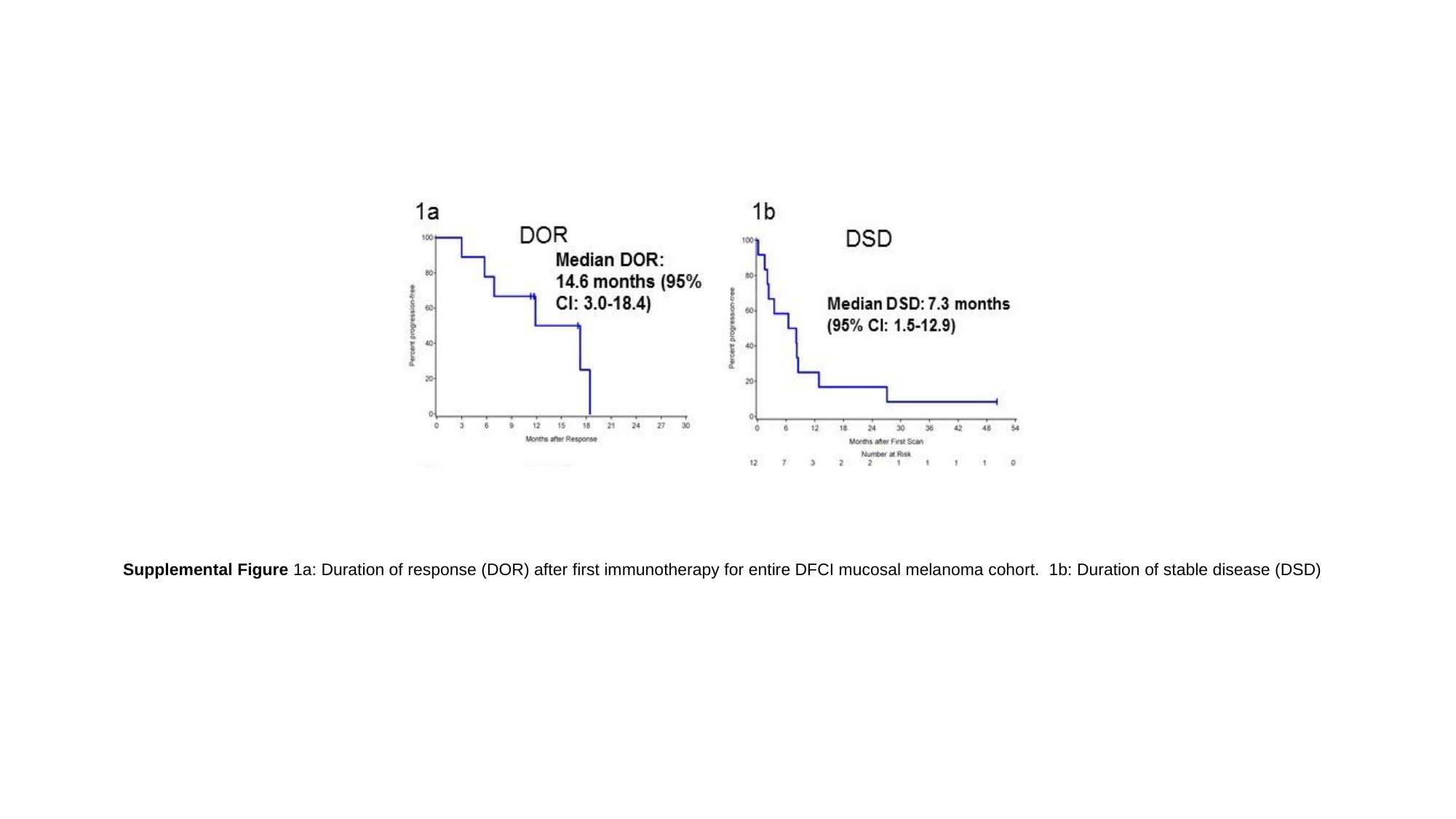

Supplemental Figure 1a: Duration of response (DOR) after first immunotherapy for entire DFCI mucosal melanoma cohort. 1b: Duration of stable disease (DSD)

## Slide 3
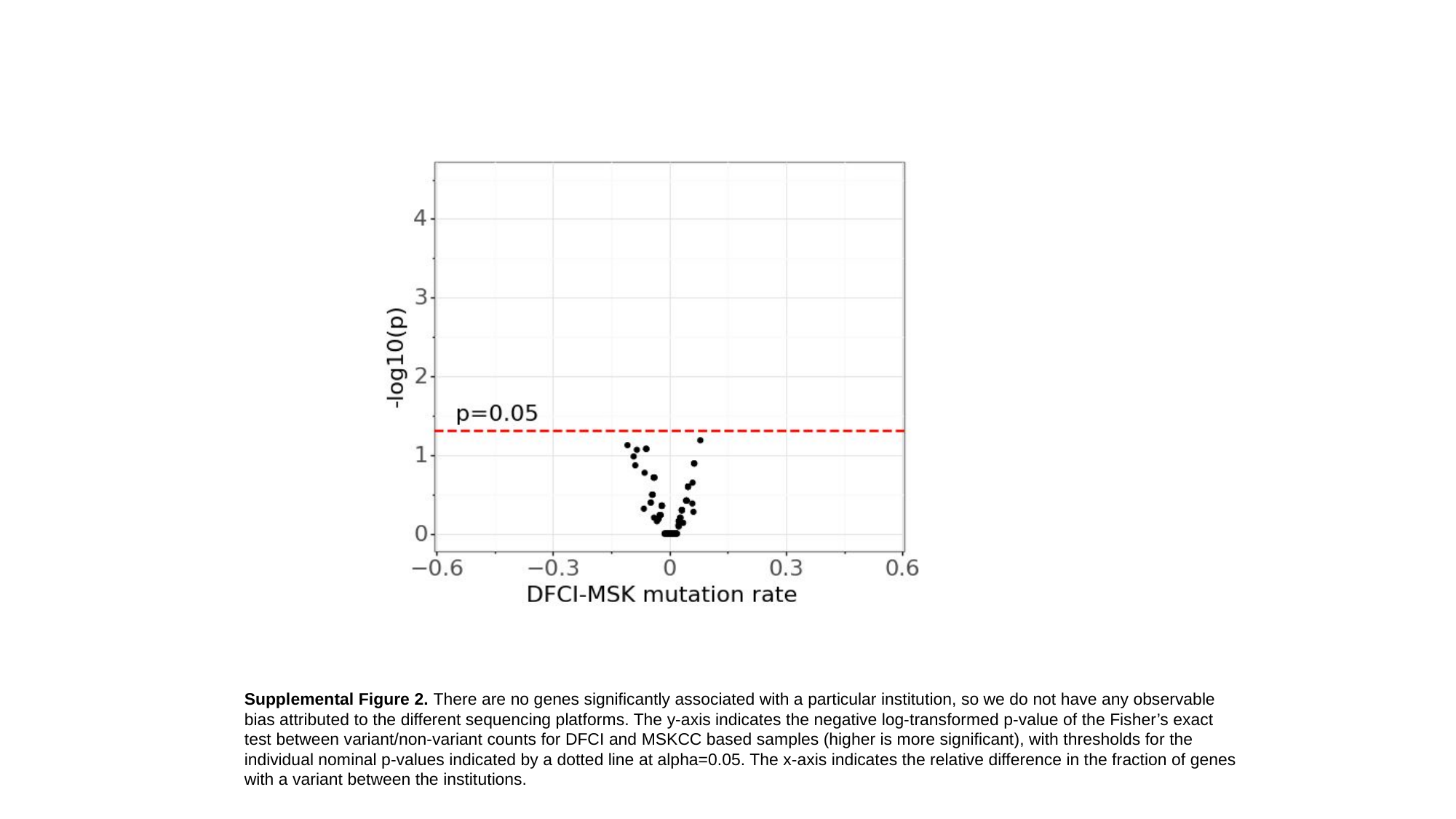

Supplemental Figure 2. There are no genes significantly associated with a particular institution, so we do not have any observable bias attributed to the different sequencing platforms. The y-axis indicates the negative log-transformed p-value of the Fisher’s exact test between variant/non-variant counts for DFCI and MSKCC based samples (higher is more significant), with thresholds for the individual nominal p-values indicated by a dotted line at alpha=0.05. The x-axis indicates the relative difference in the fraction of genes with a variant between the institutions.

## Slide 4
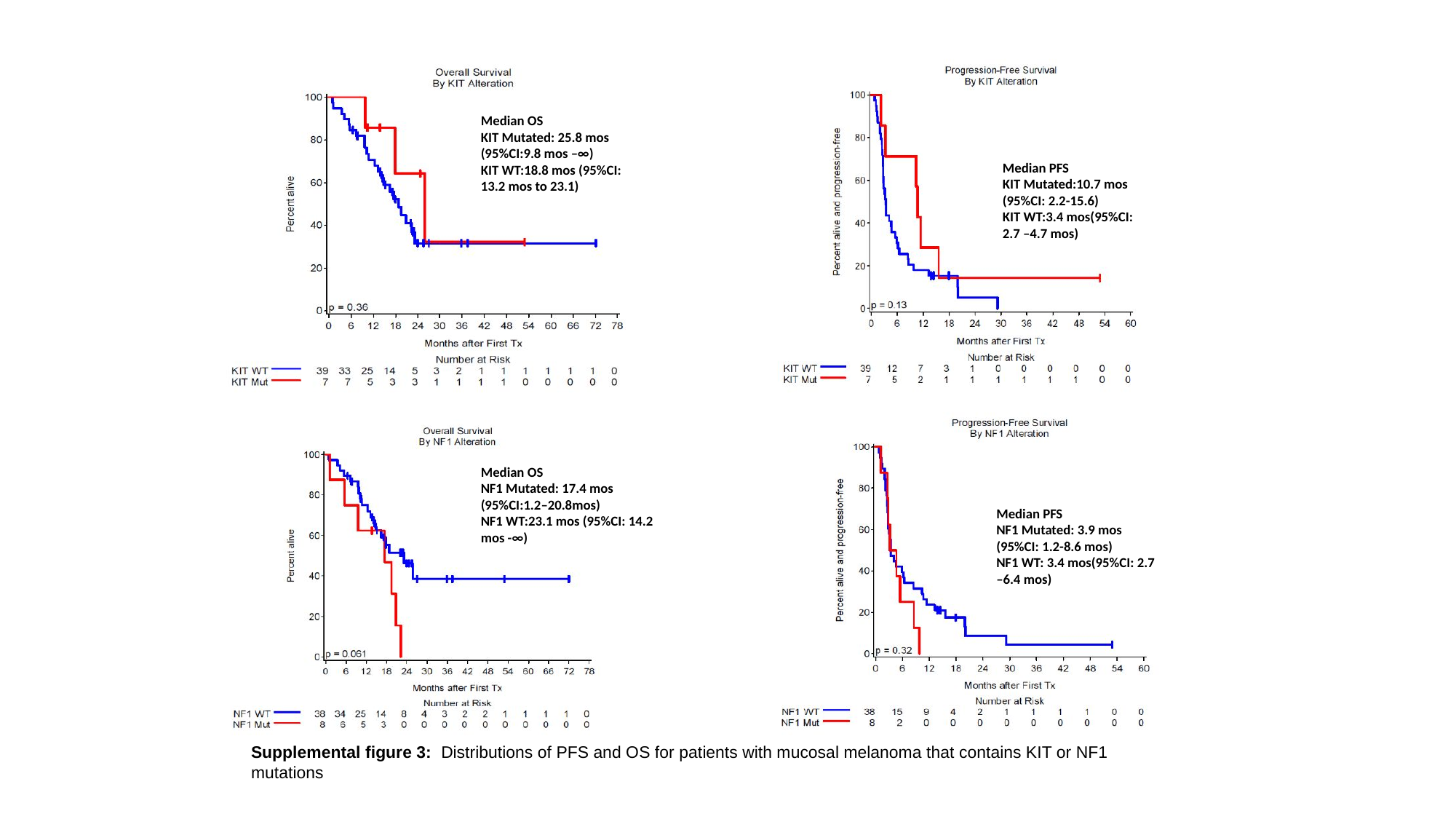

Median OS
KIT Mutated: 25.8 mos (95%CI:9.8 mos –∞)
KIT WT:18.8 mos (95%CI: 13.2 mos to 23.1)
Median PFS
KIT Mutated:10.7 mos (95%CI: 2.2-15.6)
KIT WT:3.4 mos(95%CI: 2.7 –4.7 mos)
Median OS
NF1 Mutated: 17.4 mos (95%CI:1.2–20.8mos)
NF1 WT:23.1 mos (95%CI: 14.2 mos -∞)
Median PFS
NF1 Mutated: 3.9 mos (95%CI: 1.2-8.6 mos)
NF1 WT: 3.4 mos(95%CI: 2.7 –6.4 mos)
Supplemental figure 3: Distributions of PFS and OS for patients with mucosal melanoma that contains KIT or NF1 mutations
