# Supplementary figures and images for: Characterization of genetics in patients with mucosal melanoma treated with immune checkpoint blockade
Source: Cancer Med. 2021 Mar 15;10(8):2627–35. doi: 10.1002/cam4.3789 (PMC8026918; doi:10.1002/cam4.3789)

Supplemental material

**OS**


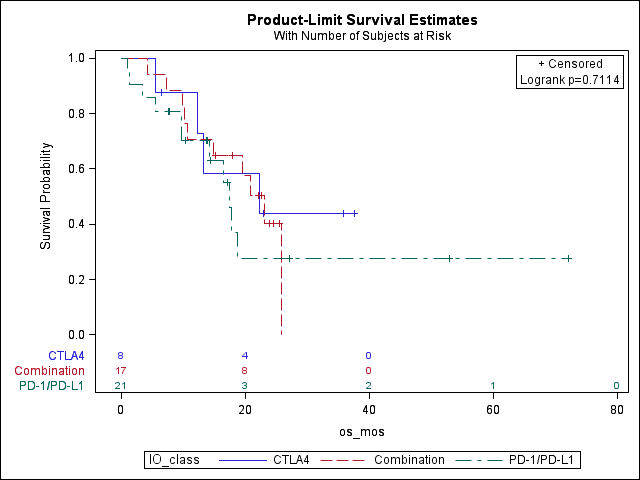


PFS


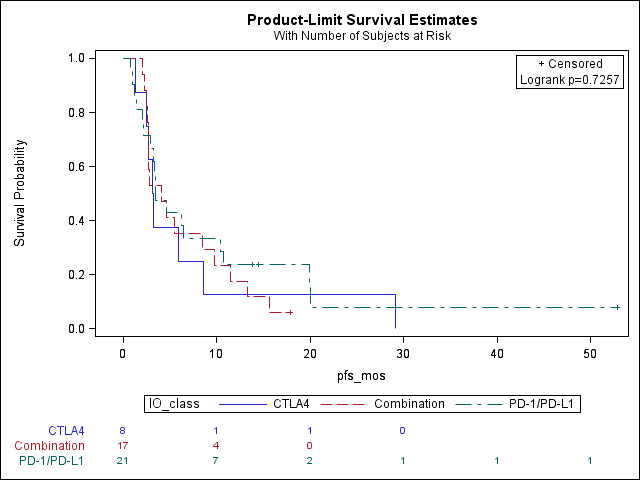


Supplemental figure 1: OS and PFS curves according to treatment.

Supplement: Supplementary file 2 — Data S1 [file CAM4-10-2627-s001.docx]
